# Trends in colorectal cancer cases at a Mexican secondary-care hospital

**DOI:** 10.3332/ecancer.2025.2040

**Published:** 2025-11-19

**Authors:** David E Gonzalez-Mendoza, Paulina P Rabago-Sanchez, Gabriel Conzuelo-Rodriguez, Angel Gomez-Villanueva

**Affiliations:** 1 Translational Research Unit, Medica Sur Clinic, Mexico City 14050, Mexico; 2 Faculty of Medicine, Autonomous University of the State of Mexico, Toluca, State of Mexico 50180, Mexico; 3 BRIMEX Clinic, Medical Center ABC, Mexico City 01120, Mexico; 4 Basic Health International, Pittsburgh, PA 15206, USA; 5 Oncology Department, Regional General Hospital 251, Mexican Social Security Institute, México City, State of Mexico 52148, Mexico; a https://orcid.org/0000-0002-3480-2621; b https://orcid.org/0000-0002-3403-6752; c https://orcid.org/0000-0002-4464-6095; d https://orcid.org/0009-0005-5996-743X

**Keywords:** colorectal cancer, incidence rates, epidemiology, secondary-care hospital

## Abstract

**Background:**

Colorectal cancer (CRC) is a major global health issue, ranking fourth in incidence and third in cancer-related deaths. In 2022, it was most prevalent in Asia, Europe and North America. Although rates in Latin America, including Mexico, are lower, they still represent a substantial public health concern. However, CRC data in Mexico are limited and outdated.

**Aim:**

This study aimed to assess the incidence trends of CRC in a secondary-level hospital in Mexico from 2011 to 2023.

**Methods:**

A retrospective analysis was conducted on 819 individuals with CRC at Regional General Hospital 251 (Mexican Social Security Institute) in Metepec, Mexico. Incidence rates were calculated per 100,000 inhabitants and stratified by sex, age group, tumour site and body mass index. Trend analysis was performed using Joinpoint regression models to estimate annual percent change (APC).

**Results:**

CRC incidence showed a significant upward trend (APC = 8.81%; *p* = 0.01) from 2011 to 2023. A one-joinpoint model revealed an increase from 2011 to 2021 (APC = 17.90%; < 0.01), followed by a sharp decrease from 2021 to 2023 (APC = −41.34%; *p* = 0.03). Males had slightly higher incidence rates than females; the ≥50 age group showed the highest burden. Over half of the individuals were diagnosed at advanced stages (III–IV), with similar trends observed across the sexes.

**Conclusion:**

CRC incidence increased significantly over the last decade, with a recent drop likely influenced by the COVID-19 pandemic. Despite some limitations, this is the first study of CRC trends at a secondary-level hospital in Mexico that underscores the need for enhanced screening and timely diagnosis strategies.

## Background

Colorectal cancer (CRC) is one of the most common types of cancer worldwide [1]. According to the Global Cancer Observatory (GLOBOCAN) (2022), CRC ranked as the fourth most frequently diagnosed cancer, with a general incidence rate (for both sexes and all ages) of 18.4 per 100,000 inhabitants [1]. Among adults (age-standardised (20–85+)), it was also the fourth most common cancer and the third leading cause of cancer-related deaths, with incidence and mortality rates of 30.5 and 13.4 per 100,000, respectively. In terms of prevalence, the continents with the highest rates of individuals with CRC in 2022 were Asia (708,879), Europe (441,579) and North America (160,897), while the lowest rates were observed in Oceania (19,503), Africa (41,445) and Latin America (including the Caribbean) (105,536) [1].

In Latin America, Mexico ranked third in CRC prevalence, with 11,933 individuals with CRC reported in 2022. The national incidence and mortality rates were 18.1 and 9.1 per 100,000 adults (20+ years), respectively, placing CRC as the fourth most common cancer in both categories [1].

According to mortality data from Mexico’s National Institute of Statistics and Geography, between 1998 and 2018, CRC mortality rates increased annually by 1.3% to 2.7%. The highest mortality was observed in northern regions, urbanised areas and populations with greater access to healthcare [2].

Despite being among the top five most frequent cancers globally and nationally, epidemiological registries for CRC in Mexico remain scarce and outdated. Few publications report epidemiological data on this disease [2–7], thus limiting the assessment of CRC trends in the Mexican population. This study aims to assess incidence trends of CRC in a secondary-level hospital in Mexico from 2011 to 2023.

## Methods

### Study design

A retrospective study was conducted from January 2011 to December 2023. Data were retrieved from electronic clinical records of individuals with primary CRC treated in the Oncology department at Regional General Hospital 251, a secondary-care hospital belonging to the Mexican Social Security Institute (IMSS, for its initials in Spanish), located in the western region of the IMSS State of Mexico. Patient information was extracted manually from the hospital electronic system (electronic clinical records) by all the authors, using a database collection form, including demographic, clinical and tumour-related variables, which are detailed in the next subsection.

### Population description

Individuals aged 20 years and older with biopsy-confirmed primary CRC were eligible for inclusion in the analysis.

CRC diagnoses were classified according to the International Classification of Diseases, Tenth Revision codes: C18 (Malignant neoplasm of colon), including subsites (cecum (C180), appendix (C181), ascending colon (C182), hepatic flexure (C183), transverse colon (C184), splenic flexure (C185), descending colon (C186), sigmoid colon (C187), overlapping colon (C188), unspecified colon (C189), C19 (Malignant neoplasm of rectosigmoid junction) and C20 (Malignant neoplasm of rectum).

Data extracted from electronic records included age, sex, weight, height, family history of cancer, comorbidities (type 2 diabetes, hypertension), clinical stage at diagnosis, tumour location (colon/rectum) and the Eastern Cooperative Oncology Group (ECOG) performance status.

### Data analysis

We carried out a descriptive analysis of the demographic characteristics of patients with primary CRC. Individuals were stratified by sex and tumour site. Incidence rates per 100,000 individuals aged 20 and older are presented. Population data were obtained on the Intranet IMSS website. The age-standardised incidence rates (ASR) were stratified by sex, from 2011 to 2023, considering the WB region: Latin America and the Caribbean, from the World Population Prospects 2024.

The overall and group-specific trends were characterised using the average annual percent change (AAPC). This summary measure proposed by Clegg *et al* [8] uses a segmented log-linear regression to detect any trend transition across the years and combine them into a unique estimate. Furthermore, AAPC reduces to the conventional annual percent change (APC) when no significant changes in rates are identified.

The analysis was performed using the National Cancer Institute’s Joinpoint Regression Program version 5.0.2 (2023) [9]. We restricted the maximum number of segments to two (i.e., one join-point) due to the relatively short time frame (13 years). For all models, the goodness-of-fit of the model with one joinpoint was compared to the fit of the null model with zero joinpoints. Significance was set at *α* = 0.05.

The assessment of quantitative variables was carried out utilising statistical tools such as Student’s* t*-test or Welch’s* t*-test (for parametric data) and the Mann-Whitney* U* test or Wilcoxon matched-pairs signed rank tests (for non-parametric data). This specific analysis was carried out in GraphPad Prism version 10.3.1.

### Ethics

This study received ethical approval from the Committee of Ethics Research 1503, attached to the IMSS (Reference: F-2024-1503-085).

## Results

Between 2011 and 2023, a total of 819 individuals with CRC were included in the study, the majority of whom were men (52.3%). The mean age was 58.3 years (SD ± 14.3) and 57.3 years (SD ± 13.8) for men and women, respectively. Family history of any cancer was positive in 400 (48.8%) subjects, whereas specific CRC history was present in 291 (35.5%). Regarding the clinical stage at diagnosis, 259 (31.6%) were reported in stage IV, 204 (24.9%) in stage III, 200 (24.4%) as an unclassified stage, 116 (14.2%) in stage II and 40 (4.9%) in stage I, while the remaining individuals had missing data regarding clinical stage. Table 1 presents the characteristics of the included individuals.

**Figure 1.**
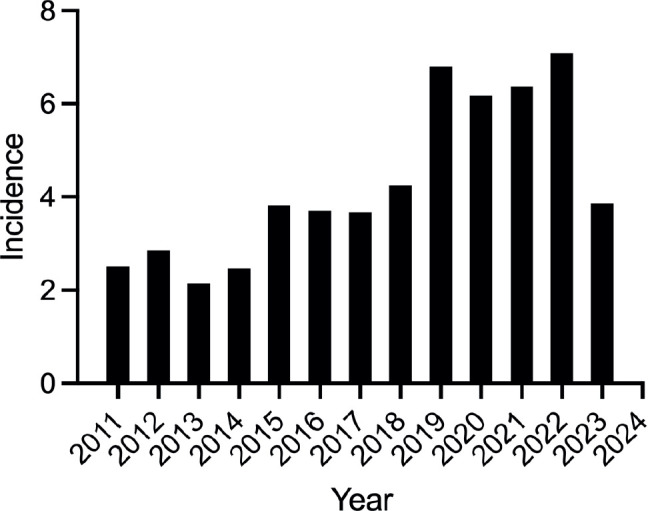
ASR of CRC per 100,000 inhabitants from 2011 to 2023 in Metepec, Mexico.

Overall, the data revealed an upward trend in newly diagnosed individuals with CRC over the years, followed by a sharp decline in 2023, reporting a median general incidence of 3.81/100,000 individuals in the general population. Figure 1 provides a graphic representation of the general population. Data regarding incidence rates are described in Table 2.

### Sex differences

When stratifying the population by tumour location, males showed a slightly higher frequency in colon (50.4%) and rectal (55.5%) cancers than females. However, this difference was not statistically significant using the chi-square test with an alpha value of 0.05 (*p* < 0.16).

Over the 13 years, men had a slightly higher median incidence rate (4.09/100,000) than women (3.85/100,000). Peak rates were also higher in men (8.95/100,000 versus 6.28/100,000), indicating a statistically significant difference between sexes (*p* = 0.01) (Table 2).

### Body mass index (BMI) associations

We divided the population based on sex and obesity status based on BMI, obtaining two groups: BMI <24.99 and BMI >25. Incidence rates were calculated, considering the specific population of men and women in the proposed period (Table 3). When comparing the trends of both groups based on BMI, a significant difference was found between the two populations for both men (*p* < 0.01) and women (*p* = 0.01). Additionally, when comparing the trends between the groups with BMI <24.99 for women and men and BMI >25 for men and women, significant differences were also identified (*p* = 0.04 and *p* = 0.04).

### Temporal trends

**Figure 2.**
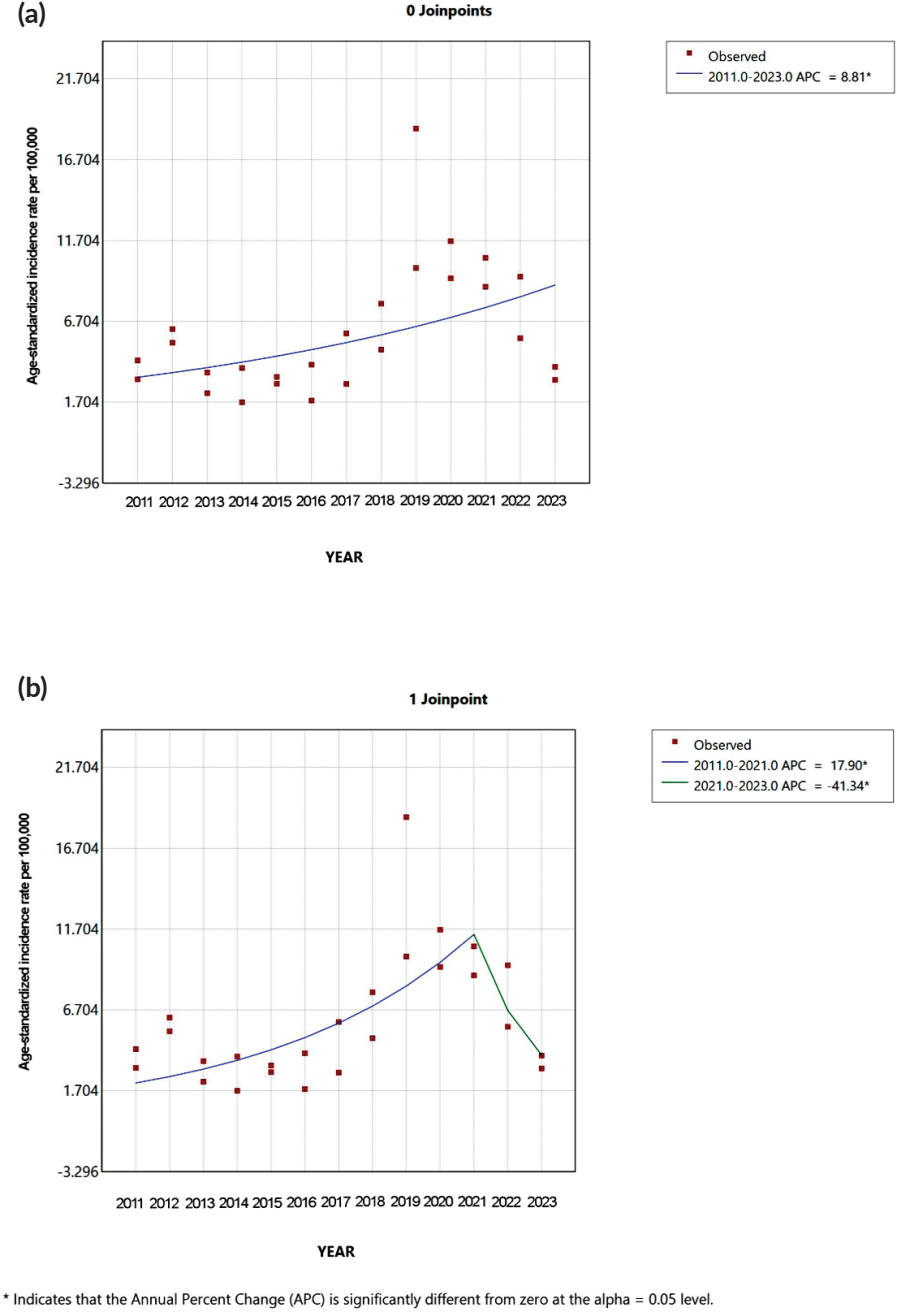
Joinpoint regression analysis of age-standardised CRC incidence rates per 100,000 inhabitants in the general population (aged 20 and older) from 2011 to 2023 in Metepec, Mexico. (a): A Model with zero joinpoints showing a statistically significant upward trend in incidence rates across the entire period (APC = 8.81%, *p *= 0.01). (b): Model with one joinpoint identifying two distinct segments: a significant increase from 2011 to 2021 (APC = 17.90%, *p *< 0.01), followed by a significant decrease from 2021 to 2023 (APC = –41.34%, *p *= 0.03). APC: Annual Percent Change. Significance set at *α* = 0.05.

Joinpoint analyses were conducted, identifying significant changes in the general population’s CRC case trends and among female and male individuals. Model 0 (0 joinpoints) showed a significant increasing trend from 2011 to 2023 with an APC of 8.81% (95% CI: 2.26 to 19.14, *p* = 0.01). In Model 1 (1 joinpoint), there were identified two segments: a significant increase from 2011 to 2021 (APC = 17.90%, 95% CI: 12.24 to 37.27, *p* < 0.01), followed by a significant decrease from 2021 to 2023 (APC = −41.34%, 95% CI: −59.14 to −3.83, *p* = 0.03) (Figure 2).

**Figure 3.**
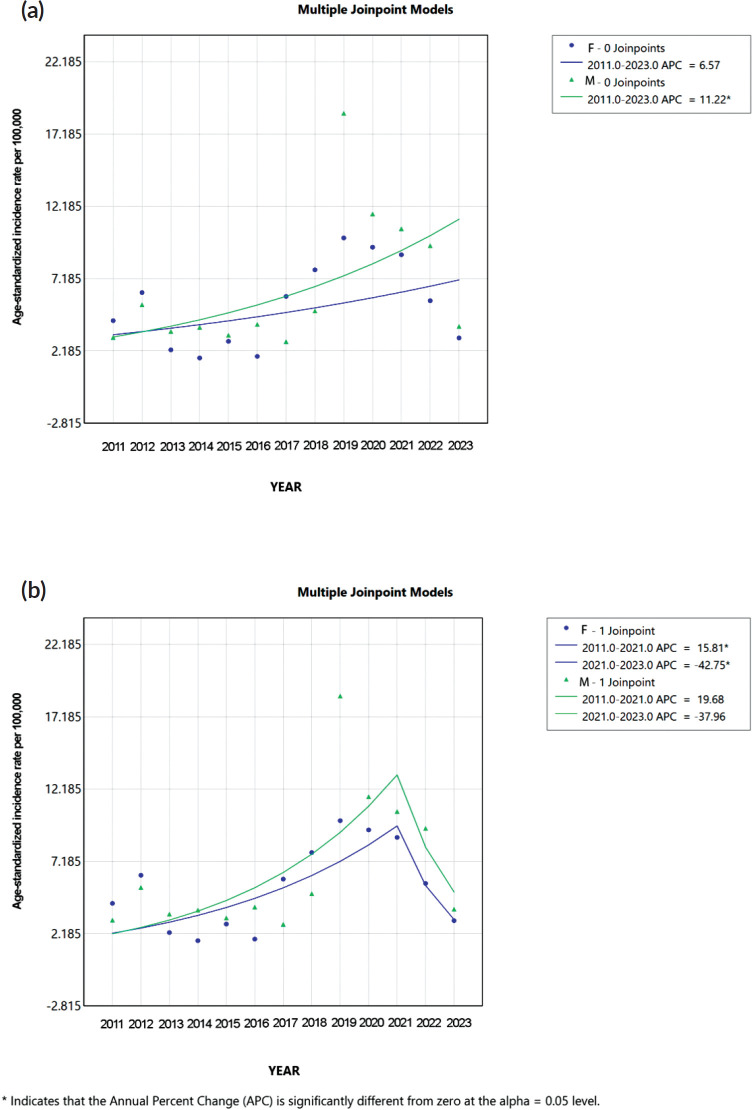
Joinpoint regression analysis of age-standardised CRC incidence rates per 100,000 population by sex in individuals aged 20 and older from 2011 to 2023 in Metepec, Mexico. Age-standardised CRC incidence rates per 100,000 population among females (blue color) and males (turquoise green) aged 20 to 80+ years from 2011 to 2023. (a): Zero-joinpoints model for both sexes. Males showed a significant increase in CRC incidence (APC = 11.22%, *p* = 0.02), while females showed a non-significant increase (APC = 6.57%, *p *= 0.17). (b): One-joinpoint model identifying two segments. Among males, a non-significant increase was observed from 2011 to 2021, followed by a non-significant decline from 2021 to 2023. Among females, the incidence increased significantly from 2011 to 2021 (APC = 15.81%, *p *< 0.01) and significantly decreased from 2021 to 2023 (APC = –42.75%, *p *= 0.05). APC: Annual Percent Change. F: Females. M: Males. Significance set at *α* = 0.05.

When analysing the trends among men and women, both sexes showed similar behaviour: In the zero joinpoints model, men’s APC was statistically significant, corresponding to 11.22% (95% CI 2.79 to 25.43, *p* = 0.02), while the women’s APC corresponded to 6.57% (95% CI −3.89 to 25.07, *p* = 0.17). Regarding the one joinpoint model, men’s APC was insignificant in the first or second trends. However, there was a change in the direction of the incidence trends. The first trend showed an increasing APC of 19.68% (95% CI −1.35 to 430.84, *p* = 0.06), while the second trend presented a decreasing APC of 37.96% (95% CI −69.50 to 24.15, *p* = 0.29). In the case of the women’s one-joinpoint model, their APCs showed significance, presenting in the first trend an increased APC of 15.81% (95% CI 9.14 to 74.68, *p* < 0.01) and in the second one a decreased APC of −42.75% (95% CI −64.19 to −0.06, *p* = 0.05) (Figure 3).

## Discussion

This study, a retrospective analysis of CRC incidence rates from 2011 to 2023 among individuals treated at a secondary-care hospital’s medical oncology department, has unveiled several significant findings. First, CRC incidence has shown a significant rise in the general population over the 13 years. Second, within this population, two distinct trends have emerged—an upward trajectory (2011–2021) followed by a decline (2021–2023). Third, incidence rates varied significantly by sex and age group (≤49 versus ≥50), with the ≥50 group showing a more pronounced trend. Fourth, the highest incidence rates occurred during the COVID-19 lockdown years, dropping afterward. These findings contribute valuable insights for national and institutional CRC surveillance strategies [1, 10, 11].

Few studies in Mexico provide epidemiological data on CRC, though international research highlights common patterns, including a slightly higher male prevalence and increased incidence in older populations [12–16].

In 2009, Charúa-Guindic *et al* [7] documented CRC incidence at a Mexico City hospital between 2015 and 2018, reporting rates of 9.7/100,000 for men and 8.2/100,000 for women—both below GLOBOCAN’s national averages (20.5/100,000 and 16/100,000, respectively) [1,7]. Similarly, our study’s highest rates [7] were also lower than national figures.

Charúa-Guindic *et al* [7] and Verastegui and Mohar [15] reported a mean age at diagnosis of over 60 years, while this study and that of Lozano-Esparza *et al* [16] reported a mean age of around 6 years younger, suggesting earlier CRC diagnosis.

Recently, other studies around the world have reported rising frequencies of individuals with CRC among younger people (under 40) [17, 18], a notable shift from its historical association with older age groups. Geographic variations in incidence also suggest possible links to environmental factors like pollution, diet and occupational exposure [19].

Globally, CRC trends vary. In the United States, by 2017, a study of 26,674 Native Americans diagnosed with colon cancer over 12 years found women were more affected than men, contrasting our male-dominated cohort (52.3%). Still, sex-based differences were nonsignificant in both studies. Joinpoint analysis supported this, aligning trends closely (Figure 3) [20]. Another variable analysed was stage at diagnosis, in which most Native American individuals with CRC were stage III at diagnosis (29%) [20]. In our study, over 50% of diagnoses occurred at advanced stages (III/IV), though staging differences by sex were not statistically significant.

Comparable results appeared in a Mexico City study of 277 individuals, where 53% had advanced CRC. That analysis also tied rural residency, weight loss, emergency surgery and rectal tumours to later-stage diagnoses [21]. Late-stage diagnosis (stages III–IV) in our cohort (> 50 %) may reflect a combination of factors: inefficiency of institutional screening programs, limited awareness among patients, insufficient recommendations from primary care providers and logistical barriers within the healthcare system [22].

Lopez-Basave *et al* [23] focused on individuals with CRC under 30 (*n* = 1,823), finding a 41.5% survival rate and 21.3% progression-free survival. Family history was reported in 27% of individuals with CRC, with rectal tumours (46%) and poorly differentiated carcinoma being the most common. Most diagnoses (62%) were stages I–III [23]. This contrasts with our study, where individuals under 50 were rarer but often advanced (III–IV). This discrepancy may reflect earlier screening in younger cohorts, indicating that early screening initiatives could be beneficial even in populations without classical risk factors or unique population traits predisposing them to early-onset CRC.

To address this, fecal immunochemical test–based (FIT) screening should be accessible to more of the population attending primary care, with significant financial and human resources. It is also critical that be complemented by culturally tailored health education and improved referral pathways to specialist services [24].

Educational initiatives aimed at both patients and frontline clinicians can improve symptom recognition and prompt action on red flag signs—such as rectal bleeding, anemia or unexplained weight loss—to reduce diagnostic delays [25, 26]. System-level enhancements such as streamlined reporting, increased training and expanded access to colonoscopy are also cardinal for closing gaps in timely detection [24]. These tools must be widely implemented and reinforced to decrease CRC diagnosis at late stages, thereby improving prognosis and post-treatment living expectancy.

While obesity is a known CRC risk factor [27], most individuals in our study were not obese: 441 had normal BMI (18.5–24.9) and 242 were overweight (25–29.9). This could relate to late diagnosis, as weight loss—a key CRC symptom—was likely prevalent, given that over half of individuals with CRC were advanced.

We performed a Joinpoint regression analysis to assess CRC frequency trends objectively, as it is a practical and widely used tool for detecting significant changes in incidence over time [28–30]. The zero joinpoint model revealed an overall upward trend in individuals with CRC. The one joinpoint model identified both an upward trend and a subsequent downward trend in recent years (2021–2023). This shift may reflect post-pandemic dynamics, [31, 32] where reduced healthcare engagement led to fewer diagnoses—potentially underestimating true incidence. If so, future years could see a rebound in cases of individuals with CRC, potentially increasing the clinical burden and demand for oncologic services in secondary-level care settings. However, alternative explanations, namely disrupted access to care, diagnostic supply shortages or reduced screening efforts, cannot be excluded.

Incidence trends were nearly identical between males and females, suggesting a homogeneous pattern. Despite this similarity, absolute incidence differed significantly by sex. In the sex-stratified one joinpoint model, the female population’s trend was statistically significant and paralleled the males’. For context, a German study noted steep declines in CRC incidence (22.4% in men, 25.5% in women), attributing this to improved screening and early detection of premalignant lesions [33]. In our population, however, the decline likely stems from other factors, as no comparable screening initiatives were implemented.

The location was selected due to its role as a regional reference hospital that reflects cancer care patterns in a densely populated urban area with limited access to early screening. This study was conducted in the Regional General Hospital 251, which is a secondary-care hospital located in Metepec, a city in the State of Mexico considered an urban town in Mexico. This hospital serves not only a part of the rural population but also a large portion of the urban one living in the State of Mexico. It accounts for more than half of the population of the State of Mexico. It acquires more relevance as the State of Mexico is the state that contains the largest population in Mexico, with 16.9 million people living in that state, based on national registries.[34]

During the COVID-19 pandemic, Regional General Hospital 251 was converted into a designated ‘COVID-19 hospital,’ prioritising the management of patients with suspected SARS-CoV-2 infection [35]. This transformation, which disrupted services, may be a potential cause of delayed care for chronic and non-communicable diseases, potentially affecting access to timely diagnostic services across the semi-rural and urban communities the hospital serves.

Despite these disruptions, our study found an increase in CRC diagnoses during the pandemic years (2019–2022). This trend could reflect several interacting factors, including increased patient awareness of symptoms, changes in healthcare-seeking behaviour or shifts in referral dynamics from primary care. It is also possible that some of these cases represent delayed diagnoses accumulated from previous months when access to care was more restricted.

While these are plausible hypotheses, the available data do not allow us to determine whether this apparent increase reflects an actual rise in incidence or a rebound effect due to diagnostic delays. Further studies are needed to investigate the underlying causes and confirm or refute this observation.

The observed increase in CRC incidence in the years preceding the COVID-19 pandemic (2011–2019) may reflect a combination of demographic and lifestyle shifts in Mexico—including an aging population, dietary westernisation, increased sedentary behaviour, obesity and diabetes—factors that have been associated with higher CRC risk [36].

Conversely, the apparent decline in CRC incidence during 2020–2023 could not represent an actual reduction in disease burden. Instead, it may reflect pandemic-related disruptions in cancer detection and diagnosis. Multiple international studies documented dramatic drops in cancer screenings—especially colonoscopy—and reduced new cancer diagnoses during early 2020, with declines of up to 50% compared to pre-pandemic periods [37–40].

These interruptions were attributed to the suspension of screening programs, reallocation of healthcare resources to COVID-19, reduced patient attendance due to fear and limited access to diagnostic services [36]. Studies from the USA and UK indicate that incidence rates did not fully rebound in 2021—suggesting a backlog of undetected cases rather than a permanent decline in incidence [36, 41].

Therefore, the decline observed in our cohort after 2020 may primarily represent underdiagnosis and delayed detection rather than a genuine reduction in CRC incidence. It is possible that a rebound in cases—or an increase in diagnoses at more advanced stages—could emerge as healthcare services stabilise. Continued monitoring is therefore essential to distinguish between temporary diagnostic disruption and actual epidemiological trends.

Mexico currently does not have an organised, population-based CRC screening program, despite rising incidence and mortality rates. National clinical guidelines recommend CRC screening with FIT in individuals aged ≥ 50. Still, these are limited to pilot initiatives or opportunistic screening in tertiary centers that are not systematically implemented at the regional or national level [42, 43].

Local studies, such as the one performed in Veracruz using FIT reported high detection rates of premalignant lesions (33%) [43]. These findings demonstrate the feasibility and potential yield of the screening based on FIT in Mexican populations. In this context, our study’s findings support the urgent need for localised screening strategies. The evidence highlights the value of implementing regionally tailored, FIT-based screening programs to detect CRC at earlier stages, especially in under-resourced areas like those served by secondary-level institutions similar to ours.

While this study focuses on a Mexican population, its implications extend globally. CRC is a global public health concern, with low and middle-income countries experiencing rising incidence due to epidemiological transitions [1, 31]. Our research findings on diagnostic delays, advanced-stage presentations and disruptions related to the COVID-19 pandemic present challenges that have not been previously reported in other regions, offering insights for health systems with similar resource constraints [21, 22, 31]. By documenting these patterns in a setting with limited screening infrastructure, the present study contributes to the broader dialogue on equitable cancer control strategies.

This is the first published incidence trend study of its kind conducted at a secondary-level hospital affiliated with the IMSS in the State of Mexico. However, several limitations were encountered during the study.

First, due to the small study population size (*n* = 819), it was not possible to perform an individual joinpoint regression analysis for each site (colon and rectum). This was reflected in highly pronounced variations from the limited data, which prevented the analysis of trends. As a result, it was decided to combine both locations as CRC to increase the sample size and thus provide greater strength to the analysis.

Second, being a retrospective study involving a review of medical oncology patient records, we found missing data in several files, which prevented a more detailed analysis using additional variables.

Third, when it comes to bias, we recognised several bias points: selection bias may arise from the single-center design, which only included the IMSS population from the western delegation of the State of Mexico; therefore, the results cannot be generalised to the entire state and other healthcare systems; diagnostic delays, particularly during the COVID-19 pandemic, could have led to underreporting of cases in 2021–2023, masking actual incidence trends; and referral bias is another concern, as our hospital’s role as a regional reference center may disproportionately attract advanced-stage cases. Nevertheless, these findings provide preliminary data that can inform future multicenter or population-based research.

## Conclusion

CRC incidence rose significantly overall, with similar trends in both sexes. Adults over 50 had the highest rates, and while incidence differed between genders, patterns of progression were comparable. The recent decline may reflect pandemic-related disruptions (e.g., reduced healthcare access and screening delays). Future research should monitor trend reversals and explore links to demographic, social and disease-specific risk factors.

## List of abbreviations

APC, Annual percent change; BMI, Body Mass Index; CRC, Colorectal cancer; ECOG, Eastern Cooperative Oncology Group; GLOBOCAN, Global Cancer Observatory; IMSS, Mexican Social Security Institute.

## Conflicts of interest

The authors declare that they have no conflicts of interest.

## Funding

The authors declare that they have no known competing financial interests or personal relationships that could have appeared to influence the work reported in this paper.

## Author contributions

David E Gonzalez-Mendoza: Conceptualisation, Investigation, Writing - Original Draft.

Paulina P Rabago-Sanchez: Investigation, Writing - Original Draft.

Gabriel Conzuelo-Rodriguez: Conceptualisation, Writing - Review and Editing, Supervision.

Angel Gomez-Villanueva: Conceptualisation, Resources, Writing - Review and Editing, Supervision.

## Figures and Tables

**Table 1.** Clinical and demographic characteristics of individuals diagnosed with CRC at Regional General Hospital 251, Metepec, Mexico, from 2011 to 2023.

Characteristics	Overall	Men *n* = 428 (52.3%)	Women *n* = 391 (47.7%)
Age, mean years ± SD	57.99 ± 14.07	58.26 ± 14.28	57.31 ± 13.82
Weight, Median (IQR)	61 (54–70)	66 (59.0–74.5)	58 (51–65)
BMI, Median (IQR)	23.95 (21.50–27.05)	24.2 (21.77–26.92)	23.6 (21.37–27.34)
Relatives with cancer	291	134 (46.1)	157 (53.9)
Comorbidities, *n* (%)	343	-	-
Diabetes type 2	139	77 (55.4)	62 (44.6)
Hypertension	204	125 (61.3)	79 (38.7)
Cancer site	-	-	-
Colon	520	262	258
Rectum	299	166	133
Clinical stage, *n* (%)	609	-	-
I	40	14 (35.0)	26 (65.0)
II	116	64 (55.2)	52 (44.8)
III	204	98 (48.0)	106 (52.0)
IV	259	146 (56.4)	113 (43.6)
ECOG, *n* (%)	589	-	-
0	95	48 (50.5)	47 (49.5)
1	327	170 (52.0)	157 (48.0)
2	111	71 (64.0)	40 (36.0)
3	49	26 (53.1)	23 (46.9)
4	7	4 (57.1)	3 (42.9)

ECOG: Eastern Cooperative Oncology Groupy scale; IQR: interquartile range; SD: standard deviation

**Table 2.** Annual ASR of CRC per 100,000 inhabitants in the general population, stratified by sex, from 2011 to 2023 in Metepec, Mexico.

	Men		Women	
Year	Individuals	Incidence	Individuals	Incidence
2011	16	3.08	13	2.05
2012	16	3.08	17	2.68
2013	14	2.56	12	1.8
2014	18	3.17	13	1.89
2015	24	4.09	25	3.57
2016	30	4.94	18	2.61
2017	22	3.37	29	3.92
2018	28	4.09	34	4.38
2019	56	7.85	48	5.9
2020	53	7.41	42	5.11
2021	48	6.5	53	6.26
2022	71	8.95	50	5.48
2023	32	3.85	37	3.85

**Table 3.** ASR of CRC per 100,000 individuals, stratified by sex and BMI category (<24.99 and ≥25), from 2011 to 2023 in Metepec, Mexico.

	Men		Women	
Year	BMI <24.99	BMI >25	BMI <24.99	BMI >25
2011	1.5	1.5	1.1	0.9
2012	1.9	1.2	2.0	0.5
2013	0.9	1.1	1.0	0.6
2014	2.3	0.7	1.5	0.4
2015	2.2	1.7	2.1	1.4
2016	2.6	2.0	1.3	0.9
2017	1.8	1.4	2.0	1.5
2018	2.2	1.8	1.8	2.4
2019	4.9	2.7	3.2	2.5
2020	4.3	2.7	2.8	2.2
2021	3.2	3.1	3.8	2.5
2022	5.4	2.9	3.8	1.4
2023	2.4	1.2	2.2	1.7

BMI: Body Mass Index
